# Evaluation of treatment planning system accuracy in estimating the stopping‐power ratio of immobilization devices for proton therapy

**DOI:** 10.1002/acm2.13831

**Published:** 2023-01-02

**Authors:** Kai Jiang, Michael MacFarlane, Sina Mossahebi, Mark J. Zakhary

**Affiliations:** ^1^ Department of Radiation Oncology University of Maryland School of Medicine Baltimore Maryland USA

**Keywords:** immobilization, proton radiotherapy, stopping power ratio, treatment planning system, water equivalent thickness

## Abstract

**Purpose:**

To assess treatment planning system (TPS) accuracy in estimating the stopping‐power ratio (SPR) of immobilization devices commonly used in proton therapy and to evaluate the dosimetric effect of SPR estimation error for a set of clinical treatment plans.

**Methods:**

Computed tomography scans of selected clinical immobilization devices were acquired. Then, the water‐equivalent thickness (WET) and SPR values of these devices based on the scans were estimated in a commercial TPS. The reference SPR of each device was measured using a multilayer ion chamber (MLIC), and the differences between measured and TPS‐estimated SPRs were calculated. These findings were utilized to calculate corrected dose distributions of 15 clinical proton plans for three treatment sites: extremity, abdomen, and head‐and‐neck. The original and corrected dose distributions were compared using a set of target and organs‐at‐risk (OARs) dose–volume histogram (DVH) parameters.

**Results:**

On average, the TPS‐estimated SPR was 19.5% lower (range, −35.1% to 0.2%) than the MLIC‐measured SPR. Due to the relatively low density of most immobilization devices used, the WET error was typically <1 mm, but up to 2.2 mm in certain devices. Overriding the SPR of the immobilization devices to the measured values did not result in significant changes in the DVH metrics of targets and most OARs. However, some critical OARs showed noticeable changes of up to 6.7% in maximum dose.

**Conclusions:**

The TPS tends to underestimate the SPR of selected proton immobilization devices by an average of about 20%, but this does not induce major WET errors because of the low density of the devices. The dosimetric effect of this SPR error was negligible for most treatment sites, although the maximum dose of a few OARs exhibited noticeable variations.

## 1 INTRODUCTION

In recent years, we have witnessed a rapid rise in the number of proton centers, and proton therapy has become more popular and accessible for treating cancers.^1^, ^2^, ^3^ The use of the Bragg peak affords proton therapy certain advantages over photon therapy, such as the lack of exit dose and reduction in integral dose. However, a prerequisite for utilizing such an advantage is accurate placement of the Bragg peaks within targets while avoiding organs‐at‐risk (OARs). Overranging of proton beams by a few millimeters can result in increases in dose to OARs distal to the tumor, and a similar magnitude of underranging can result in unacceptable undercoverage of the distal portion of the target.^4^ As a result of proton range uncertainty, a beam‐specific margin (typically between ±3.5% and ±5% of the proton range) based on the anatomy traversed by the proton beams is routinely utilized in robust optimization to ensure adequate target coverage and OAR sparing.^5^

In proton therapy, the proton range is governed by the treatment planning system (TPS)‐estimated stopping‐power ratio (SPR) calculated based on the computed tomography (CT) scan. Such estimation relies on a CT calibration curve that translates Hounsfield units (HUs) to SPR or mass density. This curve is typically generated using CT scans of tissue‐equivalent materials with known density and elemental composition.^5^ However, it is unclear whether such a curve can be used to accurately estimate the SPR of the vast number of non‐tissue‐equivalent synthetic materials used in immobilization devices,^6^, ^7^ which play a critical role in patient immobilization and reproducible positioning in proton therapy.^8^, ^9^ Because fields traversing immobilization devices are routinely used, significant inaccuracy in estimation of their water‐equivalent thickness (WET) or SPR would cause systematic errors in the proton range in the vicinity of the target. Resultantly, the American Association of Physicists in Medicine Task Group 176 recommended that the TPS‐calculated SPR should be validated with measurement during commissioning.^6^

Few reports have compared TPS‐estimated SPR and its ground truth by measurement. Wroe et al. investigated treatment couches as well as a bite block insert, compared measured to TPS‐modeled WET values, and reported a maximum discrepancy <1 mm.^10^ Fellin and coworkers expanded the list of devices by including new treatment couches, thermoplastic masks, and headrests.^11^ They found a good agreement between measured and TPS‐predicted WET for most devices. Despite these findings, many commonly used immobilization devices have not been investigated for SPR modeling accuracy. Further, it remains unclear whether inaccurate SPR estimation will have any dosimetric impact on clinical proton plans.

In this study, we aimed to assess in our clinic the accuracy of TPS‐estimated WET and SPR of selected immobilization devices potentially traversed by the proton beam. Measured WET values were compared to those predicted by the TPS from CT images acquired using different clinical CT protocols. In addition, we investigated the dosimetric impact of inaccuracy in TPS‐estimated SPR on clinical plans.

## 2 METHODS

### 2.1 Immobilization devices

Selected proton therapy immobilization devices used in our clinic were investigated, including three QFix MOLDCARE cushions (QFix; Avondale, PA), two CIVCO Vac‐Lok bags (CIVCO; Coralville, IA), one Exafast dental putty (GC America Inc.; Alsip, IL), one Sage bite block (Sage Products LLC; Cary, IL), and one kVue BoS (base of skull) insert (QFix; Avondale, PA). The QFix MOLDCARE cushion is composed of a soft fabric bag containing expanded polystyrene beads coated in a moisture‐cured resin. The CIVCO Vac‐Lok bag consists of a lightweight plastic mattress filled with radiolucent polystyrene (Styrofoam) beads. The dental putty is made of vinyl polysiloxane. The bite block contains a foam block with an embedded wooden handle. The kVue BoS insert includes a semicircular head portion made of carbon fiber and a flat body portion with carbon fiber skin and foam core. In addition, one set of thin sheets (16 layers) and thick sheets (16 layers) used to keep patients comfortable during treatment were also studied.

### 2.2 CT imaging

The immobilization devices were scanned on a Siemens SOMATOM Definition Edge CT scanner (Siemens Healthineers; Erlangen, Germany). Several radiopaque ball bearings (BBs) were attached to different locations on each immobilization device for identification during the measurement of the WET in TPS or using a multilayer ionization chamber (MLIC; Giraffe, IBA Dosimetry; Schwarzenbruck, Germany). The BB locations were chosen to sample various distinct areas on each immobilization device. Clinical CT acquisition parameters were used.

### 2.3 Measurement of the ground‐truth WET

**FIGURE 1:**
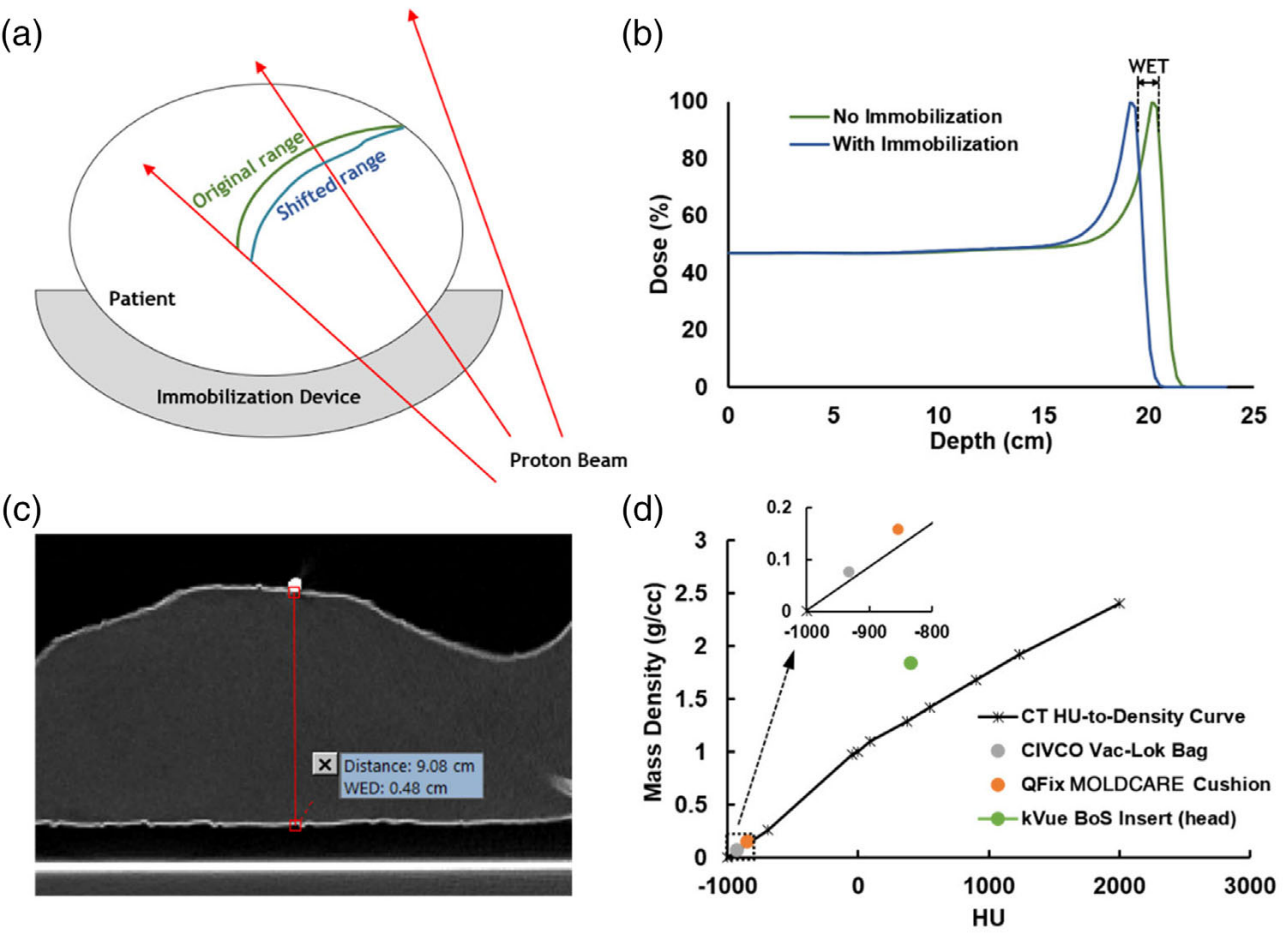
Measurement of water‐equivalent thickness (WET) and stopping‐power ratio (SPR) of immobilization devices: (a) a diagram illustrating shortened range of the Bragg peak caused by the immobilization device. Such range shift is dependent on the amount of device the beam passed through; (b) measurement of the WET of an immobilization device using the multilayer ionization chamber (MLIC) is by calculating the range shift of a monoenergetic proton beam with and without the immobilization in the beam path; (c) measurement of the WET or water‐equivalent distance (WED) and device thickness in the treatment planning system (TPS). The computed tomography (CT) slice with the largest cross section of the radiopaque bearing ball (BB), a marker of the measurement location, is used for this measurement; (d) the CT Hounsfield unit (HU)‐to‐density curve acquired by using biological samples, and scattered plot of the HU and density of three selected immobilization devices. The inset magnifies the low‐HU region. The clinical curve tends to underestimate the mass density of all three immobilization devices.

The ground‐truth WET of immobilization devices was measured using the Giraffe MLIC,^12^ comprising 180 air‐vented plane–parallel ionization chambers, with an inter‐chamber spacing of 2 mm. The MLIC was laid horizontally or vertically on the treatment couch, and a single 200‐MeV pencil beam was directed from a 270° or 0° gantry angle perpendicular to the center of the front entrance of the chamber sensitive volume. Two integrated depth–dose (IDD) curves were acquired for measurement of the WET. First, a reference IDD was obtained with the unperturbed beam directly entering the MLIC. Then, sample IDDs were acquired with an immobilization device placed on top of the chamber front entrance frame to minimize the air gap. The room lasers were used to align the immobilization device to each BB location on each device, and then the BBs were removed to avoid perturbing the beam. The WET at each location was calculated as the shift in proton range (averaging the shifts in the distal *R*80 and *R*90 values) from the reference IDD to the sample IDD (Figure 1a,b). The measured SPR at each BB location was calculated as the measured WET divided by the physical thickness determined from the CT scan, and the measured immobilization device SPR was averaged over the SPR at each BB location sampled for that device.

### 2.4 Measurement of WET and SPR in TPS

The acquired CT images were imported into the TPS (Eclipse V15.6; Varian Medical Systems; Palo Alto, CA) for the measurement of the WET and thickness. The WET and physical thickness measurements were made at each location marked with BBs on each device. To increase measurement accuracy, three measurements were made at each location, and the average value was reported. Then, the SPR was calculated as the ratio of the WET to the physical thickness. Each device SPR was calculated as the average SPR measured at each BB‐marked location on that device. Illustrations of these measurements are provided in Figure 1c.

### 2.5 Dosimetric study

**FIGURE 2:**
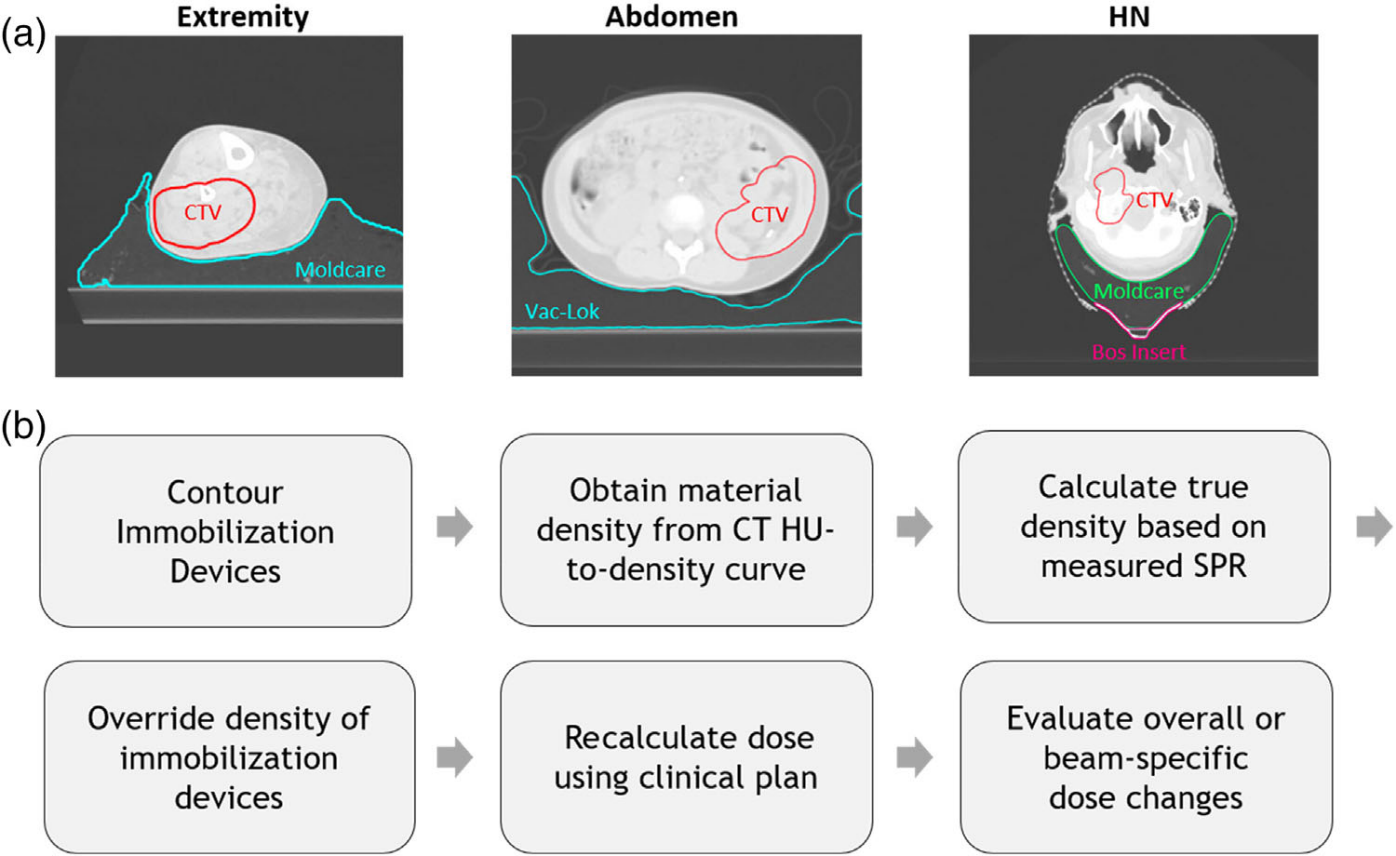
Dosimetric study sites and workflow: (a) axial computed tomography (CT) images for representative extremity, abdomen, and head‐and‐neck (HN) cases included in this study. Clinical target volumes (CTVs) and immobilization devices are outlined. Extremity patients were typically immobilized by the MOLDCARE cushion or Vac‐Lok bag, abdomen patients by the Vac‐Lok bag, and HN patients by the MOLDCARE cushion and kVue BoS insert; (b) a workflow diagram showing the steps involved in the dosimetric study. HU, Hounsfield unit; SPR, stopping‐power ratio

Based on the measurements as described, the effect of SPR estimation error by the TPS on dosimetry of clinical proton plans was studied for three immobilization devices showing the largest discrepancies between TPS‐estimated and measured SPRs: the MOLDCARE cushion, Vac‐Lok bag, and kVue BoS insert. This dosimetric study was conducted on a total of 15 clinical proton plans for 3 anatomical sites, including extremity (*n* = 5), abdomen (*n* = 5), and head‐and‐neck (HN, *n* = 5). These sites were selected as the most likely to exhibit significant portions of the treatment fields passing through the immobilization devices investigated in this report (Figure 2a). All patients receiving treatment to the HN or abdomen, as well as one patient receiving treatment to an extremity, were planned using the RayStation TPS (version 8A; RaySearch Laboratories; Stockholm, Sweden), with a Monte Carlo algorithm (uncertainty level at 0.5%) for dose calculation. The remaining four patients receiving treatment to an extremity were planned using the Eclipse TPS (version 15.6; Varian Medical Systems; Palo Alto, CA) with a pencil beam algorithm for dose calculation.

A general overview of the dosimetric workflow is shown in Figure 2b. First, each immobilization device was carefully contoured manually in the lung CT window/level (1400/−300). Owing to the relatively low SPR of the devices, it is unlikely that contouring inaccuracies would significantly affect the results of this study. The mean material mass density was obtained by converting the mean HU within the contour to mass density, using the clinical CT HU‐to‐mass density curve used for dose calculation. Second, the mass density of immobilization devices was scaled based on the difference between measured and TPS‐predicted SPRs. Then, density override was conducted by assigning either the true mass density of the material (RayStation) or the corresponding CT number (Eclipse) to the devices. Finally, a dose calculation was performed on the clinical proton plans with all beam parameters maintained, and changes in target coverage and OAR sparing compared with the originally calculated plan were evaluated.

All proton plans had multiple beams (between two and four), with each going through different areas of the immobilization devices. All proton plans were robustly optimized accounting for 3 mm (HN) or 5 mm (Abdomen, Extremity) setup uncertainty and 3.5% range uncertainty, and the plan robustness was evaluated in 12 uncertainty scenarios. In most plans, only target coverage was robustly optimized; in two cases, selected critical OARs (e.g., spinal cord, brainstem) were robustly spared as well. Dose–volume histogram (DVH) metrics used to evaluate target coverage included clinical target volume (CTV) *V*95% (volume receiving 95% of prescription dose), *D*95% (dose to 95% of volume), maximum dose, mean dose, and RTOG (Radiation Therapy Oncology Group) conformity index. CTV–DVH metrics were evaluated in the worst case robustness scenario in order to more sensitively evaluate the impact of SPR inaccuracies on target coverage. Depending on the treatment site, clinically relevant OAR DVH metrics were evaluated for the nominal plan. Finally, the beam‐specific changes in target coverage in the nominal plans were also evaluated. To obtain the beam‐specific CTV DVH metrics, an equal weight among beams was assumed.

### 2.6 Statistical analysis

All data were expressed as mean values with ranges in parentheses if applicable. For the measured WET and SPR of each immobilization device, the averaged values over multiple measured locations were used. In the dosimetric study, the Wilcoxon signed‐rank test was performed to compare DVH metrics before and after the density override of immobilization devices using MATLAB (MathWorks; Natick, MA). A *p* value <0.05 was considered statistically significant.

## 3 RESULTS

### 3.1 WET and SPR of immobilization devices

A comparison of the TPS‐estimated and MLIC‐measured SPR and WET of the immobilization devices is shown in Table 1. The TPS reliably predicted the SPR of the Exafast dental putty with a mean percentage difference of 0.2%. However, it underestimated the SPR of the other immobilization devices, with a mean of approximately −20% and a range between −12.1% and −35.1%. Despite these relatively large SPR errors, the actual WET underestimation by the TPS was within 1 mm for most devices, mainly due to their relatively low SPR values. However, the CIVCO Vac‐Lok bag and the body portion of the kVue BoS insert showed WET errors of −1.3 and −2.2 mm, respectively, mainly accounted for by their large SPR underestimation, relatively large physical thickness, or relatively high mass density. The QFix MOLDCARE cushion and the head part of the kVue BoS insert also had WET errors close to 1 mm and were also selected for investigation of the dosimetric effects of SPR inaccuracy on clinical plan quality. Figure 1d shows the CT HU‐to‐mass density curve acquired by using biological samples, and scatter plot of the HU and density of the three selected immobilization devices. The clinical curve tends to underestimate the mass density of all three immobilization devices.

**TABLE 1:** Comparison of treatment planning system (TPS)‐estimated and measured stopping‐power ratio (SPR) and water‐equivalent thickness (WET) values

Immobilization device	TPS SPR	Measured SPR	SPR %*Δ*	WET *Δ* (mm)
QFix MOLDCARE cushion	0.080 ± 0.006	0.101 ± 0.009	−20.9% ± 4.6%	−0.9 ± 0.4
CIVCO Vac‐Lok bag	0.052 ± 0.003	0.070 ± 0.002	−26.0% ± 2.9%	−1.3 ± 0.4
Exafast Dental Putty	1.519 ± 0.031	1.517 ± 0.051	0.2% ± 5.4%	0 ± 0.5
Thin sheets (16 layers)	0.171 ± 0.002	0.200 ± 0.003	−14.4% ± 0.4%	−0.2 ± 0.0
Thick sheets (16 layers)	0.129 ± 0.011	0.150 ± 0.011	−14.0% ± 1.8%	−0.5 ± 0.1
Sage Bite Block (foam)	0.212	0.241	−12.1%	−0.4
Sage Bite Block (handle)	0.364	0.485	−24.9%	−0.3
kVue BoS Insert (head)	0.790	1.100	−28.2%	−0.9
kVue BoS Insert (body)	0.122	0.189	−35.1%	−2.2

*Note*: Results for devices with more than one sample are shown as mean ± standard deviation. A single value is shown for devices with a single sample. %*Δ* = percent difference; *Δ* = difference.

### 3.2 Effects of SPR inaccuracy on clinical plans

**FIGURE 3:**
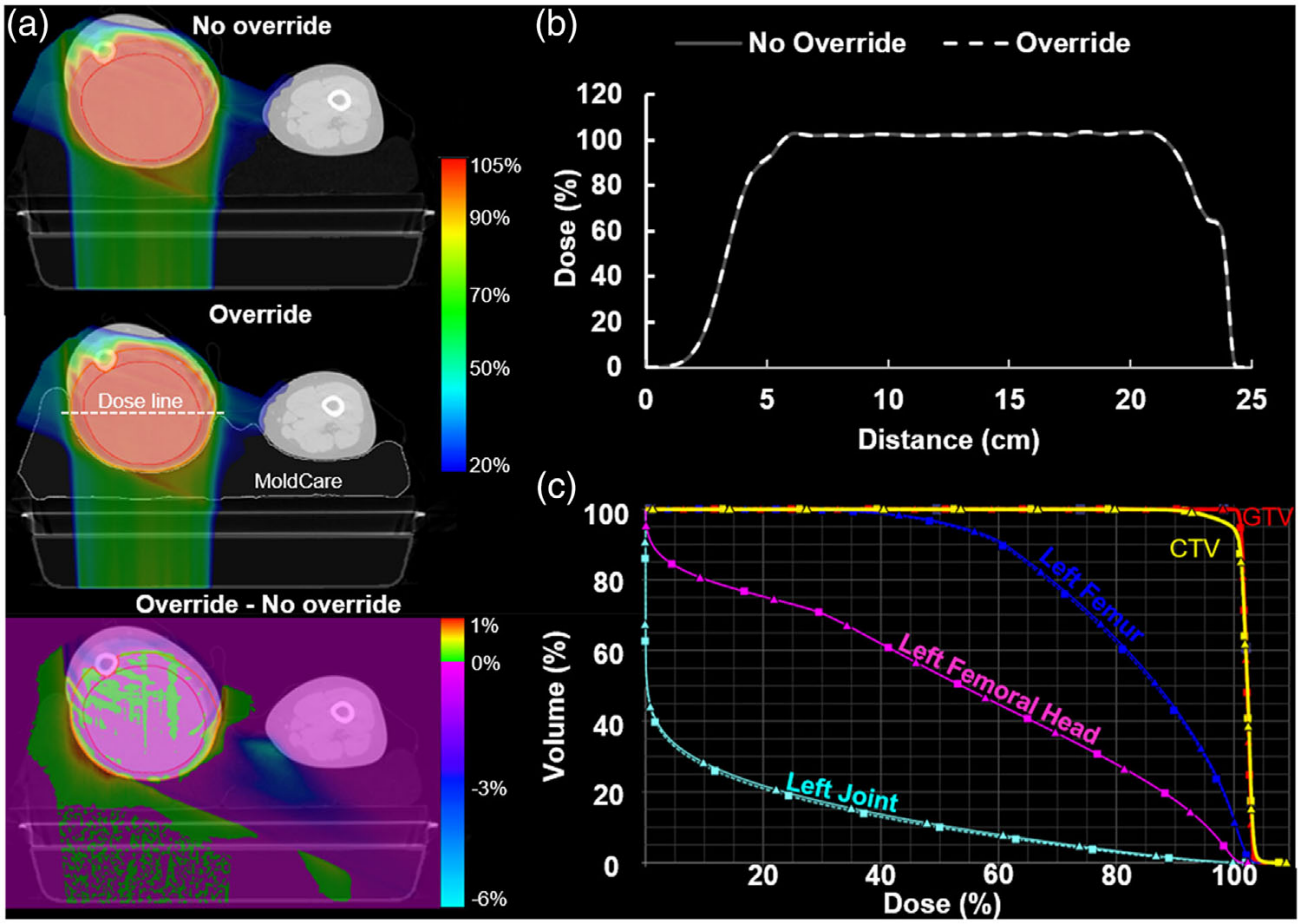
Representative dosimetric analysis of the effect of stopping‐power ratio (SPR) inaccuracy on treatment plan quality of a leg sarcoma plan: (a) an axial slice of the dose distribution on the clinically treated plan (top) and the recalculated plan with the MOLDCARE cushion overridden to the measured SPR value (middle). The gross tumor volume (GTV) and clinical target volume (CTV) are outlined in the red and orange contours, respectively. The Eclipse‐provided dose‐difference map is also shown (bottom). Minimal dose changes occurred within the target volume, but dose changes between −6% and 1% can be observed outside of the target; (b) line dose profiles through the center of the CTV (a) for both plans, demonstrating close agreement; (c) dose–volume histogram (DVH) plot of targets and relevant organ at risks (OARs), demonstrating minimal dosimetric difference between the two plans.

An extremity plan demonstrating the effect of SPR inaccuracy on overall plan quality is shown in Figure 3. A QFix MOLDCARE cushion was used to immobilize the patient's leg. One anterior–oblique beam and a second posterior beam were used to treat the leg sarcoma. Representative planar dose distributions prior to and after density override of the cushion, as well as a dose difference map (Figure 3a) demonstrate no significant changes in dose coverage of the CTV outlined in the orange contour. Line dose profiles (Figure 3b) and DVH curves of targets and OARs (Figure 3c) confirm that the effect on plan dose is minimal.

**FIGURE 4:**
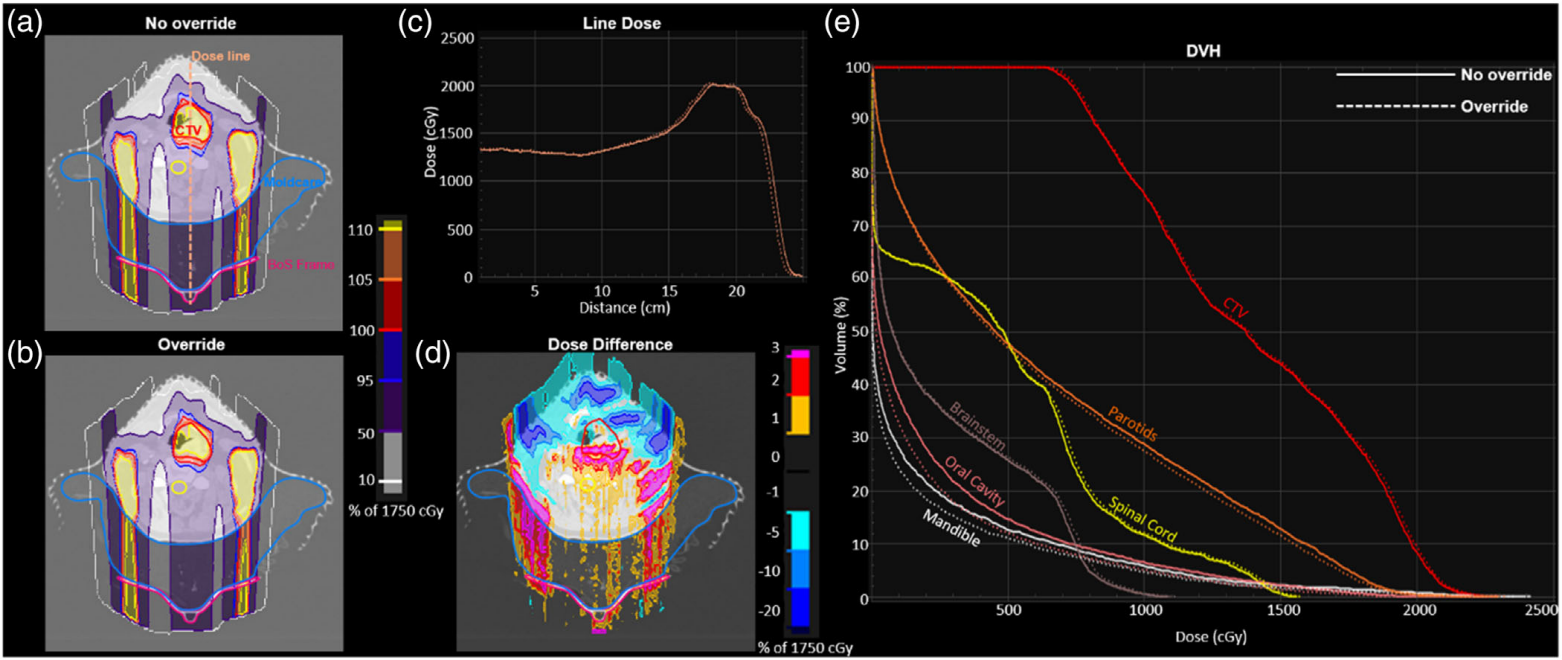
Beam‐specific dosimetric effect of overriding a MOLDCARE cushion and a BoS insert used to immobilize a patient for treatment to the head‐and‐neck (HN) patient: (a and b) representative dose maps without (a) and with (b) the density override for a 180.1° beam. The MOLDCARE is shown in the blue contour. The BoS insert is shown in the magenta contour, and its water‐equivalent thickness (WET) error is accounted for by adding the 1‐cm layer with a density override of 0.22 g/cm^3^; (c) dose profile comparison along a line across the center of the clinical target volume (CTV), as shown in part (a); (d) dose‐difference map (with–without density override); (e) dose–volume histogram shows similar CTV coverage as well as similar dose in the parotids, spinal cord, and brainstem, but slightly lower dose in the oral cavity and mandible after density override.

In addition to overall plan quality changes, the beam‐specific effects of density override of immobilization devices were also investigated. Figure 4 shows a representative HN plan showing the beam‐specific effects of TPS‐estimated SPR inaccuracy for the posterior beam. The kVue BoS insert and QFix MOLDCARE cushion were used to immobilize the patient, and the posterior beam traversed both devices to deliver dose to the CTV. The dose distributions without and with density override on these two devices are shown in parts (a) and (b) of Figure 4, respectively. Density override resulted in a pullback of the spread‐out Bragg peak by ∼3 mm, as shown by the line dose profile in Figure 4c. As a result, dose reduction in the anterior portion (−1% to −20%) and dose escalation in the posterior portion (1%–3%) were observed in the dose‐difference map (Figure 4d). Notably, similar dose changes also appeared in the CTV, but no significant changes were observed in the quantified DVH curve (Figure 4e). The OAR doses were differentially affected depending on their location along the beam path relative to the CTV (Figure 4d), supported by the slightly higher dose to proximal OARs (e.g., spinal cord, brainstem) and lower dose to distal OARs (e.g., parotids, mandible, oral cavity), as shown in Figure 4e.

A summary of the overall effects of SPR accuracy on target coverage and OAR sparing for the three anatomical sites investigated is shown in Table 2. For all three sites, worst case CTV coverage indices, including *V*95%, *D*95%, max dose, mean dose, and conformity index, all showed minimal changes. Variations in mean values were smaller than 1%, and variations in maximal values were within 2%. Changes in nominal beam‐specific target coverage were generally larger but nonetheless modest, and although some such variations were statistically significant, they are likely clinically insignificant. Most of the investigated OAR DVH metrics showed small changes within 1% on average in nominal sparing. However, for HN plans, the density override of the head‐supporting immobilization devices caused a drop in the oral cavity max dose, with an average decrease of 1.63% and maximum decrease of 6.70%. Further, a rise in the brainstem max dose with an average increase of 1.73% and maximum increase of 4.08% was observed.

**TABLE 2:** Effects of stopping‐power ratio (SPR) inaccuracy on target coverage and organ at risk (OAR) sparing

Overall CTV coverage (worst‐case robustness scenario)					
Site	*V*95% (%)	*D*95% (%)	Max dose (%)	Mean dose (%)	Conformity index
Extremity	−0.33 (−1.03, −0.04)	−0.17 (−0.49, 0.02)	−0.18 (−0.65, 0.02)	−0.08 (−0.65, 0.34)	−0 (−0.02, 0)
HN	−0.57 (−1.79, 0.11)	−0.36 (−1.05, 0.37)	0.44 (0.11, 0.94)	0.18 (−0.10, 0.37)	0.01 (−0.02, 0.04)
Abdomen	−0.07 (−0.32, 0.06)	−0.10 (−0.26, 0.02)	0.02 (−0.18, 0.19)	−0 (−0.06, 0)	0 (0, 0.01)

Beam‐specific CTV coverage (nominal plan)					
Site	Metric	*V*95% (%)	*D*95% (%)	Max dose (%)	Mean dose (%)
Extremity	*Δ*	−0.02 (−0.1, 0.1)	−0.05 (−0.23, 0.00)*	−0.09 (−1.02, 0.30)	−0.01 (−0.1, 0.07)
	%*Δ*	−0.02 (−0.22, 0.22)	−0.15 (−0.34, 0.00)*	−0.05 (−0.62, 0.28)	−0.01 (−0.07, 0.07)
HN	*Δ*	0.03 (−0.36, 0.54)	0.19 (−0.51, 1.89)	0.76 (−2.37, 4.12)	0.16 (−0.13, 0.88)*
	%*Δ*	0.28 (−0.4, 2.73)	0.22 (−0.58, 1.58)	0.56 (−1.17, 2.21)	0.12 (−0.11, 0.71)*
Abdomen	*Δ*	0.09 (−0.16, 0.89)	0.23 (−0.19, 1.82)	−0.03 (−3.5, 4.54)	0.31 (−0.1, 3.49)
	%*Δ*	0.63 (−0.47, 8.46)	0.88 (−0.22, 6.04)	−0.00 (−1.76, 1.94)	0.25 (−0.15, 1.99)

OAR sparing (nominal plan)						
Site	DVH metric(s)					
Extremity	Weight‐bearing bone *V*50Gy (%)		Skin strip *V*20Gy (%)			
	−0.24 (−0.90, 0.00)		−0.22 (−0.40, 0.02)			
HN	Spinal cord max (%)	Oral cavity max (%)	Brainstem max (%)	Mandible max (%)	Parotids max (%)	
	0.85 (−0.38, 1.65)	−1.63 (−6.7, 0.57)	1.73 (−0.42, 4.08)	−0.14 (−1.21, 0.84)	−0.27 (−0.76, 0.09)	
Abdomen	Spinal cord max (%)	Bowel max (%)	Rectum max (%)	Bladder max (%)	Kidneys mean (%)	Stomach max (%)
	−0.32 (−0.78, 0.00)	0.44 (−0.48, 2.14)	0.10 (−0.12, 0.32)	−0.01 (−0.03, 0.00)	0.88 (−0.52, 3.17)	0.55 (0.00, 1.11)

*Note*: Data expressed as mean (range). Conformity index = volume (100% isodose)/volume (CTV). Overall, CTV coverage changes calculated as difference in worst case dose–volume histogram metrics between override and no override. For beam‐specific effects, CTV coverage changes calculated as the difference (*Δ*) and percent difference (%*Δ*) between override and no override in the nominal plan. OAR sparing changes expressed as percent change in dose by density override in the nominal plan.

Abbreviations: CTV, clinical target volume; DVH, dose–volume histogram; HN, head and neck.

^*^ *p* < 0.05 comparing DVH metrics with and without density override.

## 4 DISCUSSION

SPR estimation uncertainty in proton therapy can arise from a number of sources, including CT imaging, stoichiometric formula parametrization, mean excitation energy determination, and tissue composition.^13^, ^14^, ^15^ The last of these sources of uncertainty is of particular note in investigating the SPR modeling of immobilization devices. Materials used to construct immobilization devices are typically synthetic and of low density, features highly desirable for minimizing the perturbation of proton beams. However, the composition of these devices differs substantially from the tissue‐mimicking materials used to derive the CT HU‐to‐SPR curves, which can lead to SPR estimation error. In this study, we found this underestimation to be about −20% for the devices investigated. Despite the large SPR underestimation, the relatively low density and/or small thickness still ensured an overall WET error of <1 mm in most devices, with a few exceptions in which large thicknesses (e.g., CIVCO Vac‐Lok bag) or relatively high densities (kVue BoS insert) caused WET errors close to or even larger than 1 mm. These outliers prompted a thorough investigation of the dosimetric impact of these errors on target coverage and OAR sparing in clinical proton plans.

The inaccuracy of TPS modeling of the immobilization devices was not found to lead to significant dosimetric changes in the targets and OARs for the treatment sites and plans investigated. Clinical proton plans are typically robustly optimized and contain multiple beams with different gantry angles to achieve robust target coverage, which minimizes the impact of immobilization WET estimation error.^3^, ^16^, ^17^ However, even worst case coverage on CTV targets, accounting for range and setup uncertainties which could be realized during patient treatment, were minimally affected in this study. Beams penetrating through large cross sections of the immobilization devices tend to be more impacted by such inaccuracy, as suggested by changes in OAR maximum dose by a few percent.

When opening a new proton therapy facility, evaluating the various sources of proton range uncertainty is a critical safety task. Understanding the magnitude of immobilization device WET uncertainty is a part of that undertaking. Based on this study, our clinic has elected to accept the native TPS estimation of immobilization WET without modification in the majority of cases. However, this evaluation should be taken on by a proton therapy facility's team based on the immobilization devices, treatment site distribution, planning techniques, and other factors specific to that clinic, all of which will impact the magnitude and thus the clinical significance of TPS immobilization WET estimation error.

## 5 CONCLUSIONS

In conclusion, although the TPS systematically underestimates SPR for most immobilization devices by about 20% for most devices, the low WET values of these devices result in minimal changes in the target coverage of clinical proton plans. The maximum dose to critical OARs, however, may change up to a few percent. When critical OARs are abutting the target structures and a trade‐off is made between target coverage and OAR sparing, consideration of the impact of dosimetric uncertainty from inaccurate modeling of the immobilization devices is recommended.

## AUTHOR CONTRIBUTION

Mark J. Zakhary conceived of the project. Kai Jiang, Sina Mossahebi, and Mark J. Zakhary designed the study and performed the measurements. Kai Jiang, Michael MacFarlane, and Mark J. Zakhary performed the data analysis. Kai Jiang and Mark J. Zakhary drafted the manuscript. Kai Jiang, Michael MacFarlane, Sina Mossahebi, and Mark J. Zakhary reviewed, revised, and approved the manuscript.

## CONFLICTS OF INTEREST

No conflicts of interest.
